# Machine learning for early screening of influenza A-associated invasive pulmonary aspergillosis in hospitalized patients: a real-world study

**DOI:** 10.3389/fcimb.2026.1896920

**Published:** 2026-07-09

**Authors:** Yiping Wang, Chunxiang Liu, Min Cao, Xingkai Chen, Ming Qian, Yewei Chen, Shuting Li, Hui Yang, Jinping Zhang

**Affiliations:** 1 Department of Pharmacy, Nanjing Drum Tower Hospital, China Pharmaceutical University, Nanjing, China; 2 Department of Pharmacy, Nanjing Drum Tower Hospital, Nanjing, China; 3 Department of Pharmacy Administration, The People’s Hospital of Wu Hai, Wuhai, China; 4 Department of Respiratory and Critical Care Medicine, Nanjing Drum Tower Hospital, Nanjing, China; 5 Nanjing Drum Tower Hospital, Nanjing Drum Tower Hospital Clinical College, Nanjing University of Chinese Medicine, Nanjing, China

**Keywords:** early screening, influenza A, invasive pulmonary aspergillosis, machine learning, predictive model

## Abstract

**Background:**

Influenza A-associated invasive pulmonary aspergillosis (IAPA) is a severe fungal complication with high mortality, while early identification remains difficult because of nonspecific clinical manifestations. This study aimed to develop and validate a machine learning (ML) model for early screening of IAPA in hospitalized influenza A patients.

**Methods:**

This retrospective single-center study enrolled 234 hospitalized influenza A patients from January 2023 to December 2025, including 59 patients with IPA. Eligible patients were randomly divided into a training cohort (70%) for model development and a validation cohort (30%) for internal validation. Univariate and multivariate logistic regression analyses were performed to identify independent predictors of IAPA. Five machine learning algorithms, including Logistic Regression (LR), Random Forest (RF), Support Vector Machine (SVM), XGBoost, and LightGBM, were constructed and compared to identify the most clinically applicable model for early screening.

**Results:**

Multivariate logistic regression identified seven independent predictors of IAPA, including smoking history, autoimmune disease, fibrinogen level, lymphocyte count, hemoglobin level, cumulative systemic corticosteroid dose, and corticosteroid treatment course of 8–28 days. Among the evaluated algorithms, LightGBM demonstrated the highest sensitivity (0.76) in the validation cohort and was considered the most suitable model for early screening. The LightGBM model achieved an AUC of 0.800 (95% CI, 0.714–0.886), with a specificity of 0.69 and an accuracy of 0.68.

**Conclusion:**

LightGBM serves as a robust early-warning tool for identifying influenza A patients at high risk of IAPA. Utilizing routinely available clinical data, this model facilitates bedside risk stratification and early diagnostic intervention.

## Highlights

- A machine learning-based predictive framework was developed and validated to facilitate early identification of invasive pulmonary aspergillosis (IPA) in hospitalized influenza A patients.
- Seven clinical predictors were identified to be independent risk factors for IAPA, including smoking history, autoimmune disease, cumulative corticosteroid dose, corticosteroid treatment course of 8–28 days, and specific inflammatory and hematologic markers (fibrinogen, lymphocytes, and hemoglobin).
- Among five evaluated algorithms, the LightGBM model demonstrated the highest sensitivity (0.76) in the validation cohort, prioritizing the reduction of false negatives essential for early screening.
- The LightGBM model utilizes parameters readily obtainable from routine clinical practice, enabling bedside risk stratification and intensified surveillance before the onset of overt aspergillus infection.

## 1 Introduction

Invasive pulmonary aspergillosis (IPA) is a life-threatening fungal infection traditionally associated with immunocompromised hosts; however, it is increasingly recognized in patients without classical immunosuppressive conditions (Tudesq et al., 2019). Influenza, especially type A, has been identified as a potent independent risk factor of secondary fungal infections (Shi et al., 2022; Feys et al., 2022; Schauwvlieghe et al., 2018). Mechanistically, influenza-induced epithelial injury and immune dysregulation foster a permissive microenvironment for fungal colonization (Major et al., 2023), driving the pathogenesis of influenza-associated invasive pulmonary aspergillosis (IAPA). However, a major clinical challenge lies in differentiating IAPA from severe viral pneumonia, given their nearly identical radiological and symptomatic profiles (Verweij et al., 2020). Consequently, delayed initiation of appropriate antifungal therapy are frequently observed and are strongly associated with increased mortality risk, underscoring an urgent need for improved early detection and risk stratification strategies.

Several investigations have explored the potential risk factors associated with IAPA, including host-related conditions (e.g., sex, chronic lung disease, smoking, and autoimmune disorders), immunological markers (e.g., CD4^+^T cell, lymphopenia, and fibrinogen levels), and treatment-related variables (e.g., corticosteroid exposure) (Shi et al., 2022; Fernandes et al., 2022; Fortún, 2022; Liu et al., 2021). The combination of a positive galactomannan assay in serum or bronchoalveolar lavage fluid (BAL), elevated β-D-glucan, and an exaggerated inflammatory response (e.g., high IL-6) is suggestive of early fungal invasion (Hamam et al., 2021; Dai et al., 2021; Robinson, 2022). Nevertheless, most prior studies have been descriptive or small-scale retrospective analyses, with incomplete and limited inclusion of clinical parameters, and lack integration of these variables into a practical, evidence-based risk prediction framework. To date, no validated model is available for predicting IAPA prior to the onset of overt infection, constituting a critical gap in current clinical management and anti-fungal stewardship practices.

The present study aimed to develop and validate a robust, data-driven predictive model grounded in clinical practice for early identification of influenza A patients at high risk of IPA. By integrating routinely available clinical and laboratory variables, this model offers a practical tool for bedside risk stratification and decision support. The results may offer new insights into the pathophysiological mechanisms linking influenza A and invasive fungal infections, while providing a foundation for future multi-center validation and model optimization.

## 2 Methods

### 2.1 Study design and setting

This was a single-center, retrospective observational study conducted at Nanjing Drum Tower Hospital (Nanjing, China). The Electronic Medical Record (EMR) dataset was obtained from the HIS system. Hospitalized patients (≥ 18 yr) were admitted to the study with laboratory-confirmed influenza A infection between January 2023 and December 2025. The study was approved by the Ethics Review Boards of Nanjing Drum Tower Hospital (Approval No. 2025-0842-02), and the requirement for informed consent was waived due to the retrospective nature of the study. All patient data were anonymized prior to analysis to ensure confidentiality.

### 2.2 Study population

Hospitalized patients were admitted to the study with laboratory-confirmed influenza A virus infection by nasopharyngeal swab antigen test or RT-PCR and complete clinical data available, including baseline characteristics, laboratory results, imaging findings, treatment information, and clinical outcomes. Participants were excluded based on the following criteria: (1) co-infection with other respiratory viruses, (2) long-term use of antifungal agents, (3) expected survival of less than 72 hours (e.g., terminal illness), (4) aspergillus infection diagnosed prior to the influenza A confirmation, (5) incomplete medical records or missing critical lack of clinical data, (6) pregnancy or lactation, (7) Agranulocytosis/Granulocytopenia, (8) HIV-positive. In total, 234 patients diagnosed with influenza A were screened for inclusion in the study, comprising 175 non-IPA patients and 59 IPA patients (57 proven and probable IPA infection, 2 possible IPA infection). IPA cases were defined by the 2020 European Organization for Research on Treatment of Cancer (EORTC) and Mycology Study Group (MSG) consensus definitions. Diagnosis of IPA required evidence of Aspergillus spp. infection from microbiological testing (e.g., positive sputum culture, galactomannan antigen or antigen detected in plasma, serum or BAL, and exclusion of alternative etiologies.

### 2.3 Data collection

All clinical data were obtained from the HIS system and laboratory information system. Data collection was conducted independently by two investigators, with any discrepancies resolved through joint review. Predictor variables were selected based on a comprehensive literature review regarding factors influencing influenza A. The extracted variables encompassed demographic information—such as age, sex, body mass index (BMI), smoking history, and alcohol consumption—as well as underlying medical conditions, including chronic pulmonary diseases (e.g., chronic obstructive pulmonary disease (COPD), asthma, bronchiectasis, interstitial lung disease), autoimmune disorders, diabetes mellitus, malignancy, and chronic renal disease. Clinical features at admission, including vital signs and symptoms such as cough, expectoration, chest tightness, wheezing, fever, and dyspnea, were also recorded. Laboratory parameters consisted of hematologic indices (white blood cell count, lymphocyte count, hemoglobin, platelet count), inflammatory markers (C-reactive protein and procalcitonin), coagulation profiles (fibrinogen, D-dimer, prothrombin time), and biochemical markers reflecting liver and renal function along with serum albumin levels. Treatment-related factors involved the administration of systemic corticosteroids (cumulative dose before IPA diagnosis), use of broad-spectrum antibiotics before IPA diagnosis, and the need for mechanical ventilation. Clinical outcomes assessed included the occurrence of IAPA, length of hospital stay, and in-hospital mortality. Prior to statistical analysis, all data underwent rigorous verification to ensure accuracy and completeness.

### 2.4 Data preprocessing and cohort division

Data preprocessing was performed to ensure the completeness, accuracy, and consistency of all variables prior to statistical and modeling analyses. The preprocessing procedures were conducted primarily using SPSS Statistics version 27.0.1 (IBM Corp., Armonk, NY, USA).

Data integrity was assessed by examining missing values and outliers. For continuous variables with missing rates below 10%, imputation was performed using either the median or mean value, depending on the distribution pattern. For categorical variables with missing rates below 10%, missing entries were replaced with the mode. Cases with missing rates exceeding 10% for key variables were excluded from further analysis to maintain data reliability (Afkanpour et al., 2024).

The distribution of continuous variables was examined using the GraphPad Prism software version 10.4.1 (GraphPad Software, San Diego, CA, USA) and variables were expressed as mean ± standard deviation (SD). Normality was assessed via the Shapiro–Wilk test. Variables following a normal distribution were compared between groups using the independent-samples t-test. Variables with a non-normal distribution were compared using the Mann–Whitney U test. Categorical variables were presented as counts and percentages and analyzed using the chi-square test (χ² test) in SPSS Statistics version 27.0.1.

After data cleaning and variable screening, all eligible cases were randomly divided into a training cohort (70%) and a validation cohort (30%) using a stratified random sampling method to preserve comparable proportions of IPA and non-IPA cases across datasets. This stratification ensured balanced class representation and minimized sampling bias. All randomization and preprocessing operations were implemented in SPSS Statistics version 27.0.1 and GraphPad Prism software version 10.4.1.

### 2.5 Regression analysis procedure

All statistical analyses were performed using SPSS Statistics version 27.0.1. Continuous variables and categorical variables were analyzed as described in the data preprocessing section.

Initially, univariate logistic regression analyses were conducted to identify potential factors associated with IAPA. Variables with a P value < 0.20 in the univariate analysis were considered for further evaluation. All variables identified as significant in univariate analyses were subsequently entered into a multivariate logistic regression model to determine independent predictors of IAPA. The Enter method was used, in which all candidate variables were included simultaneously to assess their independent associations. This approach avoids bias from variable selection procedures and ensures stability of parameter estimation. The multivariate analysis revealed that seven variables were independently associated with IAPA: (1) smoking history, (2) fibrinogen, (3) autoimmune disease, (4) lymphocyte count, (5) hemoglobin, (6) cumulative dose of systemic corticosteroids, and (7) duration of systemic corticosteroid therapy between 8–28 days. Odds ratios (OR) and 95% confidence intervals (CI) were calculated, and a two-tailed P value < 0.05 was considered statistically significant. Multicollinearity among these predictors was assessed using the variance inflation factor (VIF), and all variables demonstrated acceptable collinearity (VIF < 10). These seven independent predictors were subsequently incorporated into the predictive model construction for IAPA.

### 2.6 Model development

To construct a robust predictive framework for IAPA, five machine learning algorithms were employed to construct predictive models based on the identified independent predictors: Logistic Regression (LR), Random Forest (RF), Support Vector Machine (SVM), Extreme Gradient Boosting (XGBoost) and Light Gradient Boosting Machine (LightGBM). Model construction and evaluation were executed using PyCharm Community Edition 2022.2 (JetBrains, Prague, Czech Republic) with the scikit-learn, xgboost, and lightgbm libraries.

To capture comprehensive data patterns, we implemented five distinct classification algorithms ranging from linear baselines to advanced ensemble methods. LR was employed as a standard probabilistic classifier with extended iterations to ensure convergence, while SVM were utilized to address non-linear boundaries through the exploration of Linear, Polynomial, and Radial Basis Function kernels (Bloch et al., 2019). To further enhance predictive performance via ensemble learning, we deployed a RF classifier consisting of 100 unpruned decision trees alongside two gradient boosting frameworks, XGBoost and LightGBM. These boosting models were specifically configured with 100 estimators, a learning rate of 0.1, and stochastic subsampling rates of 0.8 to effectively balance model complexity and mitigate the risk of overfitting (Adnan et al., 2022).

Five-fold Grid Search Cross-Validation (GSCV) is a hyper-parameter optimization technique that systematically explores unique parameter combinations to maximize model performance. By training and evaluating multiple models through cross-validation, it identifies the specific configuration that yields the best results (Adnan et al., 2022). Model interpretability was assessed using SHapley Additive exPlanations (SHAP), which quantify the contribution of each feature to the model output (Salih et al., 2025).

### 2.7 Model evaluations

Model performance was rigorously evaluated using a clinically informed, multidimensional framework designed to prioritize early screening efficacy. Rather than relying on a single summary metric, each candidate model (LR, SVM, RF, XGBoost, and LightGBM) was systematically assessed across four core dimensions: discrimination, case-detection capacity, predictive balance, and generalization stability (Rainio et al., 2024). Discriminative ability was quantified using the area under the receiver operating characteristic curve (AUC) with 95% CIs. Overall classification accuracy and the macro-averaged F1-score were utilized to reflect global predictive balance. Given the clinical imperative of early screening to minimize missed diagnoses (false negatives), sensitivity (recall) and negative predictive value (NPV) were designated as primary indicators of case-detection capacity. Concurrently, specificity and positive predictive value (PPV) were monitored to assess the models’ ability to correctly identify true negatives and control false-alarm rates. To ensure the robustness of our findings and mitigate overfitting, all performance metrics were validated using 5-fold cross-validation.

### 2.8 Ethical approval

This study was reviewed and approved by the Ethics Committee of Nanjing Drum Tower Hospital (Approval No. 2025-0842-02). Given its retrospective nature, the requirement for individual informed consent was waived.

## 3 Results

### 3.1 Cohort description, risk factor identification, and binary logistic regression analysis

**Figure 1:**
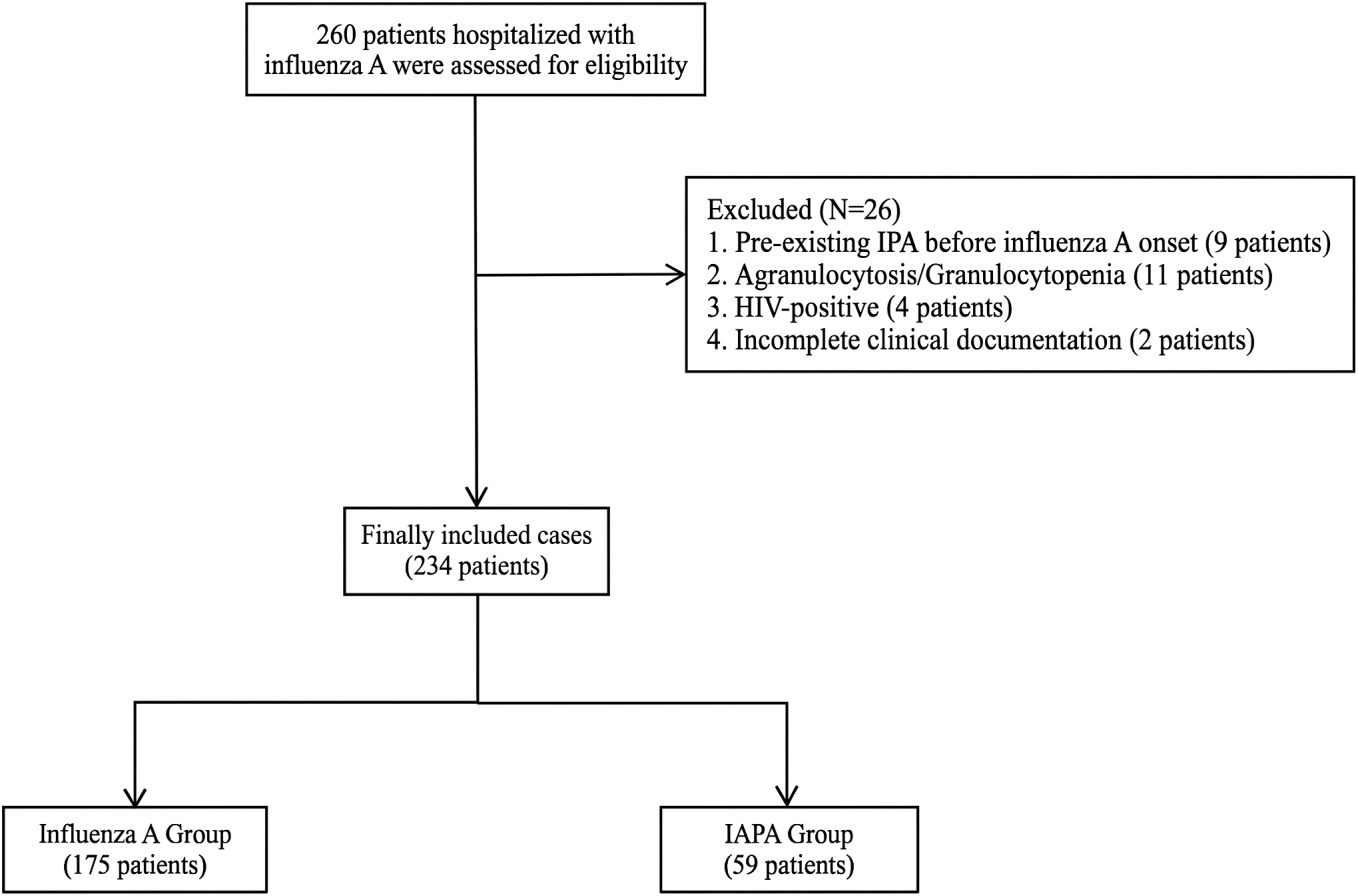
The patient flowchart with respect to inclusion and exclusion. HIV, human immunodeficiency virus.

**Figure 1** shows the patient screening and enrollment process. A total of 234 eligible hospitalized patients with influenza A screened between January 2023 and December 2025 were enrolled in the final analysis. Among this overall cohort, 59 patients (25.2%) met the dynamic diagnostic criteria for IAPA (57 proven and probable IPA infection, 2 possible IPA infection), while the remaining 175 patients served as non-IAPA controls (**Supplementary Table 1**). The discovery cohort training set consisted of 155 samples (38 IPA, 117 non-IPA), and the test set consisted of 79 samples (21 IPA, 58 non-IPA) (**Supplementary Table 2**). Baseline comparisons between IPA and non-IPA groups in the training set are presented in **Supplementary Table 3**. Of these, twenty-two variables were entered into a binary logistic regression analysis (**Supplementary Table 4**), and 7 predictors were ultimately identified for model development (**Table 1**). Analysis revealed several clinical and laboratory variables significantly associated with the outcome (all P < 0.05), encompassing smoking, autoimmune diseases, elevated fibrinogen levels, reduced hemoglobin (Hb), decreased lymphocyte count (LYM), a glucocorticoid (GC) treatment course of 8–28 days, and cumulative GC dose—all of which emerged as risk factors.

**Table 1:** Multivariate logistic regression analysis of risk factors for IAPA.

Variables	β	SE	Wald χ²	*p* value	OR	95% C.I. for OR	
						Lower	Upper
Autoimmune diseases	1.783	0.771	5.346	0.021	5.946	1.312	26.947
Smoking	2.376	0.761	9.740	0.002	10.758	2.420	47.826
Fibrinogen	0.524	0.236	4.922	0.027	1.688	1.063	2.681
Hb	-0.041	0.019	4.822	0.028	0.960	0.925	0.996
LYM	-1.634	0.754	4.694	0.030	0.195	0.045	0.856
Cumulative GC Dose	0.002	0.001	5.613	0.018	1.002	1.000	1.003
GC treatment Course of 8–28 days	1.627	0.632	6.635	0.010	5.090	1.476	17.556

β, β Coefficient; SE, standard error; Wald χ², wald chi-square test statistic.

### 3.2 Development and evaluation of machine learning models for early disease screening

**Figure 2:**
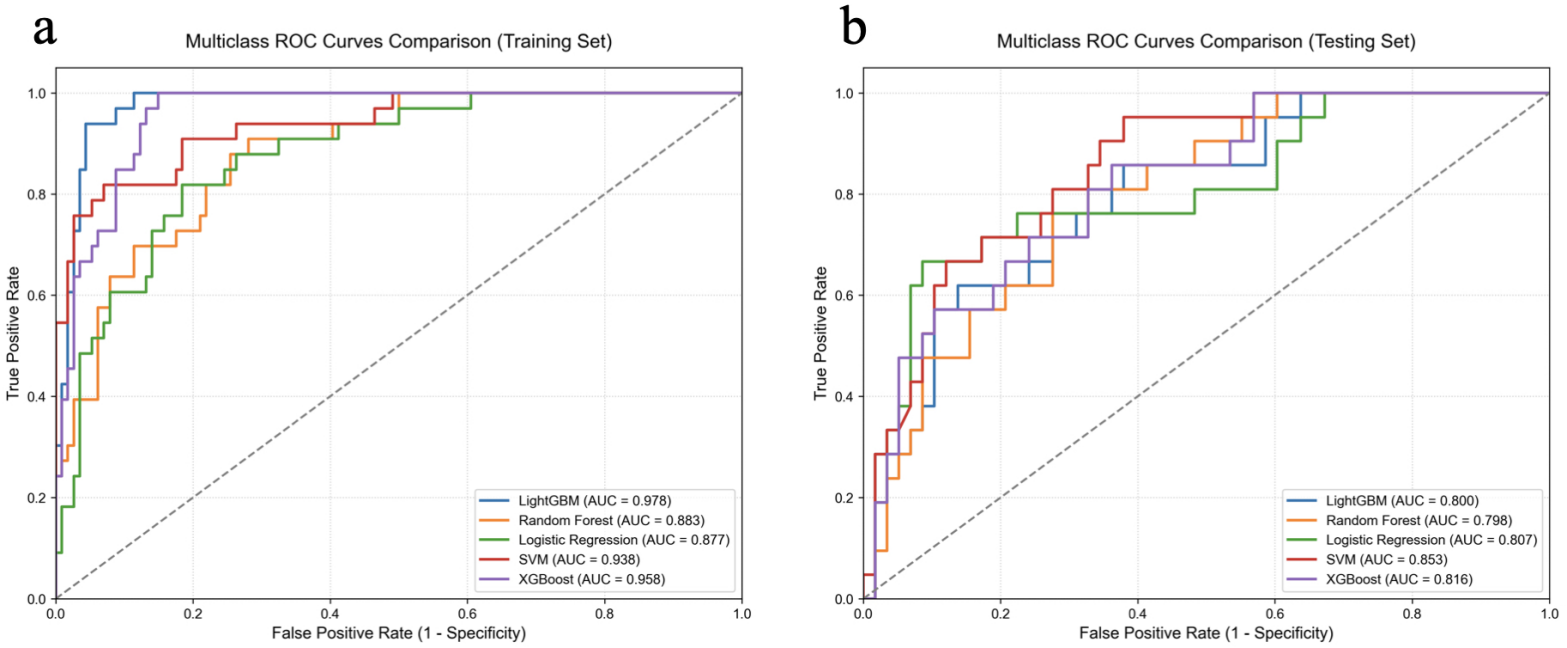
Comparison of multi-class ROC curves and AUC values across five machine learning models for the **(A)** training set and **(B)** testing set.

We trained five machine learning algorithms and systematically assessed their predictive performance on the test set. The comparative results across different models are summarized in **Table 2** and the comparison of multi-class receiver operating characteristic (ROC) curves and AUC values are shown in **Figures 2A, B**.

**Table 2:** Performance of machine learning algorithms in the outcomes of testing dataset of influenza A patients with or without IPA.

Algorithm	Accuracy	Sensitivity	Specificity	NPV	PPV	AUC [95% CI]	F1
LR	0.85	0.62	0.94	0.96	0.76	0.807 [0.689, 0.914]	0.68
SVM	0.81	0.62	0.90	0.94	0.65	0.853 [0.759, 0.931]	0.63
RF	0.80	0.48	0.92	0.93	0.67	0.798 [0.690, 0.891]	0.56
XGBoost	0.78	0.57	0.86	0.92	0.60	0.816 [0.707, 0.911]	0.59
LightGBM	0.68	0.76	0.69	0.91	0.44	0.800 [0.686, 0.902]	0.56

Based on the performance metrics in the testing dataset, the evaluative comparison revealed distinct metric trade-offs that were particularly critical in the context of early disease screening with imbalanced clinical data. While the SVM demonstrated the highest overall discriminatory power (AUC, 0.860; 95% CI, 0.782–0.938), and LR yielded the highest precision (0.76) and F1-score (0.68), both models exhibited a suboptimal sensitivity of 0.62. Given the clinical imperative of early screening—where minimizing false negatives (missed diagnoses) is prioritized over overall accuracy—LightGBM emerged as the most clinically viable candidate. It achieved the highest sensitivity (0.76), successfully identifying the largest proportion of true positive cases. Although this came at the expense of lower precision (0.44) and overall accuracy (0.68) due to the inherent class imbalance, such a trade-off is clinically justifiable for a first-line screening tool, prioritizing patient capture over the operational cost of false positives.

### 3.3 Feature importance and interpretation using SHAP

**Figure 3:**
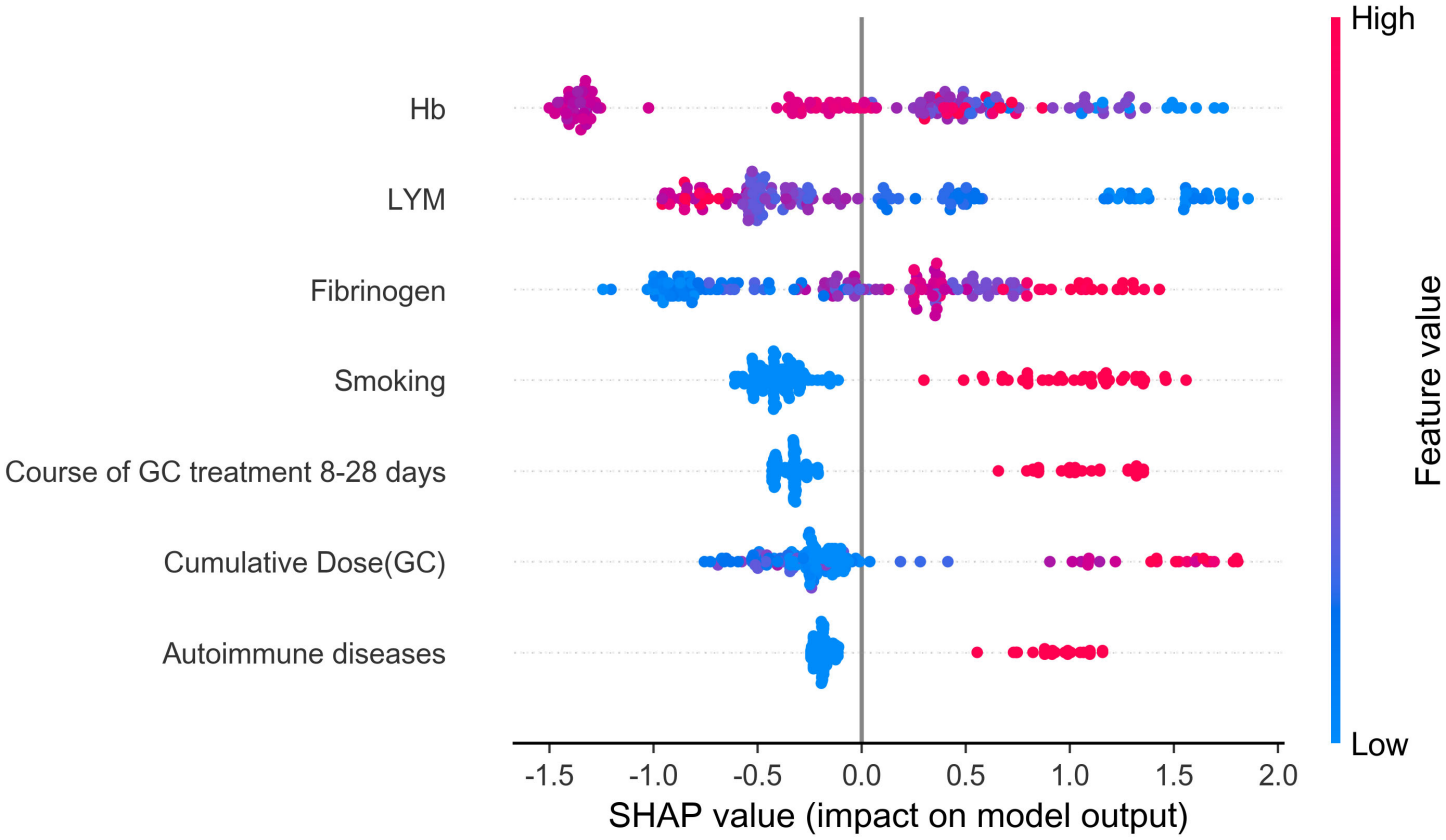
SHAP summary plot showing the contribution of each feature to the prediction of IAPA using the LightGBM model. Each dot represents an individual patient. The x-axis indicates the SHAP value (impact on model output), where positive values increase the predicted risk of IAPA and negative values decrease it. Features are ranked by importance from top to bottom. Color represents the feature value (red: high, blue: low).

The magnitude and direction of the key features’ contributions to the LightGBM model predictions were quantified and visualized via the SHAP summary plot (**Figure 3**). Elevated fibrinogen levels and an extended cumulative GC dose, inclusive of the 3-month pre-admission period, consistently yielded positive SHAP values, indicating a positive correlation with increased IAPA risk. Similarly, a history of smoking, the presence of autoimmune diseases, and a GC treatment duration of 8–28 days were positively associated with elevated risk. Conversely, LYM and Hb exhibited inverse relationships with the risk profile; lower feature values (blue dots) for both LYM and Hb were markedly clustered in the region of positive SHAP values, identifying lower lymphocyte count and decreased hemoglobin levels as notable risk factors for the development of IAPA.

### 3.4 Development of an online calculator for IAPA risk prediction

The final LightGBM model was implemented as an interactive web-based calculator, freely available at https://iapa-lightgbm-calculator.streamlit.app/, allowing users to enter predictor values and obtain the predicted probability and corresponding risk classification for hospitalized patients with IAPA.

## 4 Discussion

In this retrospective study of hospitalized patients with influenza A, we developed and validated a machine learning-based model for the early identification of individuals at high risk of IAPA. Among 22 predictive variables screened through logistic regression analysis, seven independent predictors were ultimately selected for model construction, including smoking history, autoimmune disease, fibrinogen level, lymphocyte count, hemoglobin level, cumulative systemic corticosteroid dose, and prolonged corticosteroid treatment duration. Based on comparative evaluation across five machine learning algorithms, LightGBM was considered the most clinically applicable model for early screening because it achieved the highest sensitivity in the test set. These findings suggest that routinely available clinical and laboratory variables may help identify influenza A patients at increased risk of IAPA before overt fungal infection is clinically recognized.

Previous studies on IPA have largely focused on classically immunocompromised hosts, particularly recipients of solid organ transplantation, neutropenia, patients with hematologic malignancies or hematopoietic stem cell transplantation, and individuals living with HIV (Shi et al., 2022; Schauwvlieghe et al., 2018; Donnelly et al., 2020; Peral et al., 2023; Rouzé et al., 2022; Shi et al., 2025). In contrast, more recent studies on IAPA have increasingly extended beyond these traditional high-risk populations and have often focused on patients considered immunocompetent (Chen et al., 2020; Duan et al., 2021). Against this background, we enrolled immunocompetent patients in this study. And we further included patients with autoimmune diseases who did not meet the conventional definition of severe immunosuppression. Although not classically immunocompromised, many of these patients are exposed to prolonged immunosuppressive therapy or systemic corticosteroids, which may still meaningfully impair host defense against fungal invasion. This distinction is clinically important, as previous studies have also identified autoimmune disease as a potential, and in some analyses independent, risk factor for IAPA (Arranz-Herrero et al., 2023; Valikhani et al., 2025). Therefore, by including both immunocompetent patients and those with non-severe immunosuppression, our model may better capture the real-world heterogeneity of hospitalized patients with influenza A than an approach restricted to apparently immunocompetent individuals alone.

Our finding regarding lymphocyte count is in line with prior studies suggesting that lymphopenia may be more than a marker of severe influenza; it may also reflect impaired cellular antifungal defense in patients who subsequently develop IAPA (Shi et al., 2025; Chen et al., 2020; Chao et al., 2021; Jiang et al., 2025; Fan et al., 2024; Fan et al., 2025). Available evidence suggests that influenza-associated T-cell depletion is related to apoptosis and pulmonary sequestration, while excessive type I interferon signaling can further promote lymphopenia and suppress the IL-23/Th17 axis; together, these changes may weaken antimicrobial peptide production and neutrophil recruitment that are required for antifungal clearance (Putter and Seghatchian, 2023; Salazar et al., 2022). Notably, although the mechanistic link between lymphopenia itself and IAPA remains incompletely defined in humans, current evidence supports a biologically plausible association, potentially driven by virus-induced lymphocyte apoptosis and progressive T-cell exhaustion; in this context, CD4+ T-cell depletion may impair Th1- and Th17-dependent antifungal immunity, thereby weakening local phagocyte activation, antifungal cytokine release, and control of Aspergillus hyphal growth (Chen et al., 2020; Dhume et al., 2025; Salazar et al., 2022).

Our analysis also identified smoking history as a significant contributor to IAPA risk. Chronic tobacco exposure impairs mucociliary clearance, disrupts airway epithelial integrity, and alters local innate immune responses, thereby facilitating fungal colonization and invasion (Shi et al., 2022; Derqui et al., 2022; Rudwan et al., 2026).

In addition to host baseline conditions, treatment-related exposures—specifically the duration and cumulative dose of systemic corticosteroids—were essential components of our predictive framework. Previous evidence does not support the routine use of corticosteroids in influenza pneumonia, and meta-analytic data suggest that their use is associated with higher mortality, prolonged Intensive Care Uite (ICU) stay, and increased secondary infection (Ni et al., 2019; Martin-Loeches and Torres, 2021). Nevertheless, in clinical practice, corticosteroid use is often unavoidable in patients receiving long-term or intermittent therapy for underlying diseases. In studies specifically addressing IAPA, systemic corticosteroid use before IPA diagnosis and prolonged corticosteroid exposure have also been identified as major risk factors for IPA development, even in patients without classical immunocompromising conditions (Chen et al., 2020; Chao et al., 2021). Mechanistically, corticosteroids may promote IAPA by weakening antifungal host defense in lungs already injured by influenza. They suppress macrophage, neutrophil, and T-cell responses, blunt protective cytokine signaling, and may further impair epithelial barrier function, creating a setting that favors Aspergillus adherence, persistence, and invasion (Lamoth et al., 2021). Consistent with these reports, our data showed significantly greater cumulative glucocorticoid exposure in the IPA group, and a treatment course of 8–28 days remained independently associated with IPA. This may reflect sustained corticosteroid-induced impairment of host antifungal defense, rather than the effect of a single short exposure (Ni et al., 2019).

Higher fibrinogen levels in the IPA group may reflect a more profound inflammatory and tissue-injury milieu. As an acute-phase reactant, fibrinogen usually rises in severe viral pneumonia and critical illness, and in the setting of influenza this may indicate more extensive pulmonary injury and dysregulated host inflammation (Oliva et al., 2021). Experimental and mechanistic data further suggest that Aspergillus conidia can bind to fibrinogen, while lung injury increases the exposure of fibrinogen and other extracellular matrix components on the damaged respiratory epithelium, potentially creating a microenvironment more permissive for fungal adherence and subsequent invasion (Elkhapery et al., 2025). Low hemoglobin in patients with influenza-associated fungal superinfection likely reflects a more fragile host state, marked by persistent inflammation and disordered iron metabolism, rather than serving as a specific marker of fungal infection. In this setting, impaired oxygen-carrying capacity and immune dysfunction may weaken host defense, while influenza-related epithelial injury further facilitates fungal invasion. Overall, low hemoglobin may be better interpreted as a signal of increased susceptibility to secondary fungal infection after influenza (Guo and Tong, 2021; Mohus et al., 2025).

An important component of this study was the comparison of different machine learning algorithms in relation to a clearly defined clinical objective. Although SVM achieved the highest AUC and LR showed superior overall precision and F1-score, LightGBM yielded the highest sensitivity in the test dataset. This distinction was particularly relevant because the primary purpose of the model was not to maximize overall classification accuracy, but to facilitate early identification of hospitalized influenza A patients at elevated risk for IAPA (Liu et al., 2020). In this setting, failing to identify a true high-risk patient may delay further fungal evaluation and antifungal intervention, with potentially serious consequences for prognosis. By contrast, false-positive classifications, while undesirable, are more acceptable within an initial screening framework (Zheng et al., 2020).

The lower precision observed for LightGBM should therefore be interpreted in the context of the highly imbalanced nature of the dataset rather than as a simple deficiency of model performance (Thabtah et al., 2020). When the prevalence of true positive cases is low, even a modest false-positive rate can generate a substantial number of false-positive predictions, thereby reducing precision. From a clinical standpoint, however, this trade-off is consistent with the intended role of the model. A screening-oriented tool should prioritize the capture of as many potentially at-risk patients as possible, even at the expense of over-triage. Individuals identified as high risk can then undergo more specific secondary evaluation, such as repeated fungal biomarker testing, bronchoalveolar lavage, microbiological assessment, or intensified radiologic surveillance, to distinguish true cases from false alarms (Karad et al., 2025). In this sense, the model is better understood as a sensitive early-warning instrument than as a standalone diagnostic classifier.

Model selection, however, was not based on sensitivity alone. Predictive stability across datasets was also a key consideration, particularly given the limited number of predictors and the marked class imbalance, both of which increase the risk of overfitting. For this reason, training and test performance were examined in parallel to identify models that retained their discriminative and screening capacity beyond the development dataset.

## 5 Findings

Our findings also extend the existing literature in an important way. Previous studies on IAPA have largely focused on describing incidence, mortality, and individual risk factors such as corticosteroid exposure, chronic lung disease, immune impairment, or fungal biomarkers (Shi et al., 2022; Verweij et al., 2020; Shi et al., 2025; Lu et al., 2024; Feys et al., 2024; Sedik et al., 2026; Wang et al., 2025; Su et al., 2026). In contrast, the present study integrates multiple routine clinical variables into a practical predictive framework aimed at bedside risk stratification. This may represent a meaningful step from descriptive epidemiology toward decision-support modeling. Another practical advantage is that the predictors used in our model are readily obtainable during routine hospitalization, without requiring highly specialized or invasive procedures. This feature may improve the feasibility of future implementation in real-world hospital settings, especially in situations where early bronchoscopy or repeated fungal biomarker testing is not universally available. Consistent with this objective, Aspergillus-specific diagnostic indicators, including the (1→3)-β-D-glucan (G) test, galactomannan (GM) test, and other Aspergillus-related assays, were deliberately excluded from model construction. The rationale was to preserve the model’s value for early risk stratification, enabling clinicians to identify vulnerable patients and intensify monitoring before definitive evidence of Aspergillus infection emerges.

## 6 Limitation

Several limitations of this study should be acknowledged. First, this was a retrospective, single-center study, which may introduce selection bias and limit generalizability. Second, the overall sample size was modest, and the number of IAPA cases was relatively small, which may affect the stability of model estimation and increase the risk of overfitting despite the use of internal validation. Third, the training and test cohorts were derived from the same institution, and external validation in independent multicenter datasets is still required before broader application.

## 7 Future work

Future studies should therefore focus on prospective multicenter validation of this model, ideally in larger and more diverse influenza populations. It would also be valuable to examine whether integrating dynamic laboratory trends, radiologic features, and fungal biomarkers could further improve discrimination and calibration. Ultimately, a clinically useful IAPA prediction tool should not replace diagnostic judgment, but rather assist clinicians in identifying which influenza A patients require intensified fungal surveillance and earlier diagnostic intervention.

## 8 Conclusion

We developed a machine learning-based model for early prediction of IPA in hospitalized patients with influenza A using seven routinely available clinical variables. Among the tested algorithms, LightGBM showed the greatest potential as a screening tool because of its superior sensitivity in detecting patients at elevated risk. These findings support the feasibility of data-driven early warning strategies for IAPA and provide a basis for future multicenter validation and model refinement.

## Data Availability

The original contributions presented in the study are included in the article/supplementary material. Further inquiries can be directed to the corresponding authors.
