# Supplementary material for: Machine learning for early screening of influenza A-associated invasive pulmonary aspergillosis in hospitalized patients: a real-world study
Source: Front Cell Infect Microbiol. 2026 Jul 9;16:1896920. doi: 10.3389/fcimb.2026.1896920 (PMC13391404; doi:10.3389/fcimb.2026.1896920)
Supplement: Supplementary file 1 [file Table1.docx]

**Supplementary Table 1. Comparison of Baseline Characteristics Between Control and IPA Cohorts in Influenza A Patients**

| Patient characteristics | Con set (n=175) | IPA set (n=59) | P-value |
| --- | --- | --- | --- |
| **Demographics** |  |  |  |
| Age, mean (sd) | 67.66±13.29 | 67.05±11.22 | 0.751 |
| BMI, mean (sd) | 23.2±3.38 | 22.64±3.06 | 0.019 |
| Gender, n (%) |  |  |  |
| Male | 80(45.71%) | 34(57.63%) | 0.113 |
| Female | 95(54.29%) | 25(42.37%) |  |
| **Lifestyle factors, pulmonary comorbidities and respiratory symptoms** | | | |
| Smoking, n(%) |  |  |  |
| Yes | 44(25.14%) | 21(35.59%) | 0.121 |
| No | 131(74.86%) | 38(64.41%) |  |
| Drinking, n (%) |  |  |  |
| Yes | 22(12.57%) | 13(22.03%) | 0.078 |
| No | 153(87.43%) | 46(77.97%) |  |
| Lung cancer, n(%) |  |  |  |
| Yes | 44(25.14%) | 5(8.47%) | 0.007 |
| No | 131(74.86%) | 54(91.53%) |  |
| Interstitial lung disease, n(%) |  |  |  |
| Yes | 43(24.57%) | 27(45.76%) | 0.002 |
| No | 132(75.43%) | 32(54.24%) |  |
| Pulmonary emphysema and bullae lung, n(%) |  |  |  |
| Yes | 33(18.86%) | 16(27.12%) | 0.177 |
| No | 142(81.14%) | 43(72.88%) |  |
| Bronchiectasis, n(%) |  |  |  |
| Yes | 25(14.29%) | 4(6.78%) | 0.130 |
| No | 150(85.71%) | 55(93.22%) |  |
| AECOPD, n(%) |  |  |  |
| Yes | 40(22.86%) | 14(23.73%) | 0.891 |
| No | 135(77.14%) | 45(76.27%) |  |
| Pulmonary hypertension, n(%) |  |  |  |
| Yes | 15(8.57%) | 9(15.25%) | 0.143 |
| No | 160(91.43%) | 50(84.75%) |  |
| Pleural effusion, n(%) |  |  |  |
| Yes | 29(16.57%) | 12(20.34%) | 0.510 |
| No | 146(83.43%) | 47(79.66%) |  |
| Pneumonia, n(%) |  |  |  |
| Yes | 68(38.86%) | 22(37.29%) | 0.830 |
| No | 107(61.14%) | 37(62.71%) |  |
| Severe pneumonia, n(%) |  |  |  |
| Yes | 24(13.71%) | 16(27.12%) | 0.018 |
| No | 151(86.29%) | 43(72.88%) |  |
| Cough, n(%) |  |  |  |
| Yes | 155(88.57%) | 55(93.22%) | 0.309 |
| No | 20(11.43%) | 4(6.78%) |  |
| Expectoration, n(%) |  |  |  |
| Yes | 127(72.57%) | 50(84.75%) | 0.060 |
| No | 48(27.43%) | 9(15.25%) |  |
| Chest tightness, n(%) |  |  |  |
| Yes | 68(38.86%) | 33(55.93%) | 0.022 |
| No | 107(61.14%) | 26(44.07%) |  |
| Wheeze, n(%) |  |  |  |
| Yes | 82(46.86%) | 44(74.58%) | <0.001 |
| No | 93(53.14%) | 15(25.42%) |  |
| Fever, n(%) |  |  |  |
| Yes | 109(62.29%) | 34(57.63%) | 0.526 |
| No | 66(37.71%) | 25(42.37%) |  |
| Dyspnea, n(%) |  |  |  |
| Yes | 5(2.86%) | 3(5.08%) | 0.689 |
| No | 170(97.14%) | 56(94.92%) |  |
| Heart failure, n(%) |  |  |  |
| Yes | 2(1.14%) | 2(3.39%) | 0.568 |
| No | 173(98.86%) | 57(96.61%) |  |
| Respiratory failure, n(%) |  |  |  |
| Yes | 58(33.14%) | 32(54.24%) | 0.004 |
| No | 117(66.86%) | 27(45.76%) |  |
| **Comorbidities** |  |  |  |
| History of [valve surgery](https://cncontext.com/english-chinese/translation/valvular+surgery), n(%) |  |  |  |
| Yes | 4(2.29%) | 2(3.39%) | 1.000 |
| No | 171(97.71%) | 57(96.61%) |  |
| Atrial fibrillation, n(%) |  |  |  |
| Yes | 4(2.29%) | 3(5.08%) | 0.516 |
| No | 171(97.71%) | 56(94.92%) |  |
| Coronary Heart Disease, n(%) |  |  |  |
| Yes | 9(5.14%) | 4(6.78%) | 0.884 |
| No | 166(94.86%) | 55(93.22%) |  |
| Cerebral Infarction, n(%) |  |  |  |
| Yes | 19(10.86%) | 4(6.78%) | 0.363 |
| No | 156(89.14%) | 55(93.22%) |  |
| VTE, n(%) |  |  |  |
| Yes | 2(1.14%) | 2(3.39%) | 0.568 |
| No | 173(98.86%) | 57(96.61%) |  |
| Pulmonary embolism, n(%) |  |  |  |
| Yes | 1(0.57%) | 0(0.00%) | 1.000 |
| No | 174(99.43%) | 59(100.00%) |  |
| Hypertension, n(%) |  |  |  |
| Yes | 69(39.43%) | 20(33.90%) | 0.449 |
| No | 106(60.57%) | 39(66.10%) |  |
| Diabetes, n(%) |  |  |  |
| Yes | 31(17.71%) | 18(30.51%) | 0.037 |
| No | 144(82.29%) | 41(69.49%) |  |
| Cardiovascular diseases, n(%) |  |  |  |
| Yes | 67(38.29%) | 31(52.54%) | 0.055 |
| No | 108(61.71%) | 28(47.46%) |  |
| Chronic Respiratory Diseases, n(%) |  |  |  |
| Yes | 173(98.86%) | 55(93.22%) | 0.058 |
| No | 2(1.14%) | 4(6.78%) |  |
| Chronic kidney diseases, n(%) |  |  |  |
| Yes | 21(12.00%) | 11(18.64%) | 0.199 |
| No | 154(88.00%) | 48(81.36%) |  |
| Digestive system diseases, n(%) |  |  |  |
| Yes | 9(5.14%) | 5(8.47%) | 0.538 |
| No | 166(94.86%) | 54(91.53%) |  |
| Neurological diseases, n(%) |  |  |  |
| Yes | 8(4.57%) | 1(1.69%) | 0.547 |
| No | 167(95.43%) | 58(98.31%) |  |
| Hematological diseases, n(%) |  |  |  |
| Yes | 4(2.29%) | 3(5.08%) | 0.516 |
| No | 171(97.71%) | 56(94.92%) |  |
| Autoimmune diseases, n(%) |  |  |  |
| Yes | 20(11.43%) | 20(33.90%) | <0.001 |
| No | 155(88.75%) | 56(94.92%) |  |
| Types of underlying diseases, mean (sd) | 2.26±1.16 | 2.81±1.37 | 0.003 |
| **Laboratory tests following influenza A and prior to IPA diagnosis** | | |  |
| D-Dimer, mean (sd) | 1.78±3.16 | 2.85±4.87 | 0.052 |
| INR, mean (sd) | 1.24±2.07 | 1.06±0.16 | 0.523 |
| APTT, mean (sd) | 28.39±4.11 | 27.93±4.13 | 0.461 |
| FIB, mean (sd) | 4.41±1.79 | 4.72±1.75 | 0.240 |
| BPC, mean (sd) | 202.34±103.21 | 207.2±86.53 | 0.709 |
| WBC, mean (sd) | 7.67±3.86 | 9.41±4.88 | 0.006 |
| NEU, mean (sd) | 5.83±3.61 | 7.8±5.07 | 0.001 |
| MONO, mean (sd) | 0.56±0.63 | 0.63±0.96 | 0.530 |
| EOS, mean (sd) | 0.24±0.91 | 0.36±1.71 | 0.482 |
| Hb, mean (sd) | 119.85±19.02 | 114.47±23.05 | 0.077 |
| LYM, mean (sd) | 1.22±0.7 | 0.98±0.6 | 0.019 |
| CRP, mean (sd) | 54±57.43 | 68.96±52.65 | 0.079 |
| [BLD](http://wap.medix.cn/Module/Examination/ReferenceAndSignificance/Text.aspx?code=02010000012), n(%) |  |  |  |
| Yes | 63(36.00%) | 18(30.51%) | 0.443 |
| No | 112(64.00%) | 41(69.49%) |  |
| FOB, n(%) |  |  |  |
| Yes | 53(30.29%) | 15(25.42%) | 0.477 |
| No | 122(69.71%) | 44(74.58%) |  |
| CD4＜200, n(%) |  |  |  |
| Yes | 22(12.57%) | 18(30.51%) | 0.002 |
| No | 153(87.43%) | 41(69.49%) |  |
| CD4/CD8 ratio＜1, n(%) |  |  |  |
| Yes | 37(21.14%) | 18(30.51%) | 0.142 |
| No | 138(78.86%) | 41(69.49%) |  |
| IgE＞375, n(%) |  |  |  |
| Yes | 8(4.57%) | 4(6.78%) | 0.746 |
| No | 167(95.43%) | 55(93.22%) |  |
| PCT, mean (sd) | 0.71±3.12 | 1.29±3.97 | 0.251 |
| IL-6, mean (sd) | 68.89±128.66 | 99.12±268.03 | 0.257 |
| LDH, mean (sd) | 254.7±107.66 | 293.37±121.24 | 0.022 |
| ALT, mean (sd) | 30.5±43.1 | 29.07±32.13 | 0.816 |
| AST, mean (sd) | 33.52±31.36 | 34.04±44.5 | 0.921 |
| STB, mean (sd) | 11.12±5.14 | 11.64±6.97 | 0.547 |
| Alb, mean (sd) | 34.87±4.64 | 32.78±4.69 | 0.003 |
| Cr, mean (sd) | 70.41±25.89 | 82.32±77.57 | 0.079 |
| Serum and/or BAL GM antigen >1, n(%) |  |  |  |
| Yes | 8(4.57%) | 34(57.63%) | <0.001 |
| No | 167(95.43%) | 25(42.37%) |  |
| (1→3)-β-D-glucan 0–60 pg/mL, n(%) |  |  |  |
| Yes | 151(86.29%) | 45(76.27%) | 0.071 |
| No | 24(13.71%) | 14(23.73%) |  |
| (1→3)-β-D-glucan 60–100 pg/mL, n(%) |  |  |  |
| Yes | 10(5.71%) | 2(3.39%) | 0.720 |
| No | 165(94.29%) | 57(96.61%) |  |
| (1→3)-β-D-glucan >100 pg/mL, n(%) |  |  |  |
| Yes | 14(8.00%) | 12(20.34%) | 0.009 |
| No | 161(92.00%) | 47(79.66%) |  |
| Aspergillus GM antigen >0.5, n(%) |  |  |  |
| Yes | 2(1.14%) | 7(11.86%) | 0.001 |
| No | 173(98.86%) | 52(88.14%) |  |
| Aspergillus-specific IgG 0–79 AU/mL, n(%) |  |  |  |
| Yes | 162(92.57%) | 53(89.83%) | 0.696 |
| No | 13(7.43%) | 6(10.17%) |  |
| Aspergillus-specific IgG 80–119 AU/mL, n(%) |  |  |  |
| Yes | 3(1.71%) | 3(5.08%) | 0.347 |
| No | 172(98.29%) | 56(94.92%) |  |
| Aspergillus-specific IgG >119 AU/mL, n(%) |  |  |  |
| Yes | 10(5.71%) | 3(5.08%) | 1.000 |
| No | 165(94.29%) | 56(94.92%) |  |
| **The use of wide-spectrum antibiotic before IPA diagnosis** | |  |  |
| Course of treatment, n(%) |  |  |  |
| <7 days | 29(16.57%) | 6(10.17%) | 0.233 |
| 7–14 days | 72(41.14%) | 16(27.12%) | 0.054 |
| >14 days | 64(36.57%) | 36(61.02%) | 0.001 |
| Cephalosporins, n(%) |  |  |  |
| Yes | 59(33.71%) | 25(42.37%) | 0.231 |
| No | 116(66.29%) | 34(57.63%) |  |
| Fluoroquinolones, n(%) |  |  |  |
| Yes | 93(53.14%) | 36(61.02%) | 0.293 |
| No | 82(46.86%) | 23(38.98%) |  |
| Enzyme inhibitor, n(%) |  |  |  |
| Yes | 95(54.29%) | 37(62.71%) | 0.259 |
| No | 80(45.71%) | 22(37.29%) |  |
| Carbapenem, n(%) |  |  |  |
| Yes | 25(14.29%) | 17(28.81%) | 0.012 |
| No | 150(85.71%) | 42(71.19%) |  |
| Vancomycin, Teicoplanin, Linezolid, Daptomycin, n(%) |  |  |  |
| Yes | 19(10.86%) | 11(18.64%) | 0.122 |
| No | 156(89.14%) | 48(81.36%) |  |
| Special-use antibiotics, n(%) |  |  |  |
| Yes | 13(7.43%) | 6(10.17%) | 0.696 |
| No | 162(92.57%) | 53(89.83%) |  |
| **Glucocorticoids use within three months** | |  |  |
| Cumulative Dose, mean (sd) | 187.97±331.94 | 505.02±629.73 | <0.001 |
| Course of treatment, n(%) |  |  |  |
| ≤7 days | 58(33.14%) | 15(25.42%) | 0.268 |
| 8–14 days | 20(11.43%) | 13(22.03%) | 0.043 |
| 14–28 days | 10(5.71%) | 12(20.34%) | 0.001 |
| >28 days | 11(6.29%) | 10(16.95%) | 0.013 |
| **Other medication before IPA diagnosis** | |  |  |
| The use of Bronchodilator for inhalation within a month, n(%) |  |  |  |
| Yes | 100(57.14%) | 40(67.80%) | 0.149 |
| No | 75(42.86%) | 19(32.20%) |  |
| The use of ICS within a month, n(%) |  |  |  |
| Yes | 93(53.14%) | 42(71.19%) | 0.015 |
| No | 82(46.86%) | 17(28.81%) |  |
| Immunosuppressive drugs, n(%) |  |  |  |
| Yes | 17(9.71%) | 13(22.03%) | 0.014 |
| No | 158(90.29%) | 46(77.97%) |  |
| Chemotherapy, n(%) |  |  |  |
| Yes | 34(19.43%) | 4(6.78%) | 0.023 |
| No | 141(80.57%) | 55(93.22%) |  |
| **Imaging examination** |  |  |  |
| Cavity, n(%) |  |  |  |
| Yes | 2(1.14%) | 5(8.47%) | 0.016 |
| No | 173(98.86%) | 54(91.53%) |  |
| Tree-in-bud pattern, n(%) |  |  |  |
| Yes | 40(22.86%) | 12(20.34%) | 0.687 |
| No | 135(77.14%) | 47(79.66%) |  |
| GGO, n(%) |  |  |  |
| Yes | 55(31.43%) | 14(23.73%) | 0.262 |
| No | 120(68.43%) | 45(76.27%) |  |
| Reticular shallow, n(%) |  |  |  |
| Yes | 45(25.71%) | 24(40.68%) | 0.029 |
| No | 130(74.29%) | 35(59.32%) |  |
| Consolidation, n(%) |  |  |  |
| Yes | 4(2.29%) | 2(3.39%) | 1.000 |
| No | 171(97.71%) | 57(96.61%) |  |
| Pleural effusion, n(%) |  |  |  |
| Yes | 59(33.71%) | 22(37.29%) | 0.618 |
| No | 116(66.29%) | 37(62.71%) |  |
| Mediastinal lymphadenectasis, n(%) |  |  |  |
| Yes | 74(42.29%) | 27(45.76%) | 0.641 |
| No | 101(57.71%) | 32(54.24%) |  |
| **Special medical procedures in ICU** |  |  |  |
| ICU admission, n(%) |  |  |  |
| Yes | 32(18.29%) | 20(33.90%) | 0.013 |
| No | 143(81.71%) | 56(94.92%) |  |
| Mechanical ventilation, n(%) |  |  |  |
| Yes | 22(12.57%) | 16(27.12%) | 0.009 |
| No | 153(87.43%) | 43(72.88%) |  |
| Central venous catheter, n(%) |  |  |  |
| Yes | 8(4.57%) | 7(11.86%) | 0.095 |
| No | 167(95.43%) | 52(88.14%) |  |

**Supplementary Table 2. Baseline characteristics of patients in both the Training and Test sets.**

| Patient characteristics | Training set (n=155) | Test set (n=79) | P-value |
| --- | --- | --- | --- |
| **Demographics** |  |  |  |
| Age, mean (sd) | 67.09±12.33 | 68.33±13.68 | 0.48 |
| BMI, mean (sd) | 22.98±3.96 | 23.28±4.01 | 0.95 |
| Gender |  |  |  |
| Male, n (%) | 86(55.48%) | 43(54.43%) | 0.88 |
| Female, n(%) | 69(44.52%) | 36(45.57%) |  |
| **Lifestyle factors, pulmonary comorbidities and respiratory symptoms** | | | |
| Smoking, n(%) |  |  |  |
| Yes | 46(29.68%) | 19(24.05%) | 0.36 |
| No | 109(70.32%) | 60(75.95%) |  |
| Drinking, n (%) |  |  |  |
| Yes | 23(14.84%) | 12(15.19%) | 0.94 |
| No | 132(85.16%) | 67(84.81%) |  |
| Lung cancer, n(%) |  |  |  |
| Yes | 31(20.00%) | 18(22.78%) | 0.62 |
| No | 124(80.00%) | 61(77.22%) |  |
| Interstitial lung disease, n(%) |  |  |  |
| Yes | 47(30.32%) | 23(29.11%) | 0.85 |
| No | 108(69.68%) | 56(70.89%) |  |
| Pulmonary emphysema and bullae lung, n(%) |  |  |  |
| Yes | 34(21.94%) | 16(20.25%) | 0.77 |
| No | 121(78.06%) | 63(79.75%) |  |
| Bronchiectasis, n(%) |  |  |  |
| Yes | 19(12.26%) | 10(12.66%) | 0.93 |
| No | 136(87.74%) | 69(81.34%) |  |
| AECOPD, n(%) |  |  |  |
| Yes | 35(22.58%) | 19(24.05%) | 0.80 |
| No | 120(77.42%) | 60(75.95%) |  |
| Pulmonary hypertension, n(%) |  |  |  |
| Yes | 16(10.32%) | 8(10.13%) | 0.96 |
| No | 139(89.68%) | 71(89.87%) |  |
| Pleural effusion, n(%) |  |  |  |
| Yes | 28(18.06%) | 13(16.46%) | 0.76 |
| No | 127(81.94%) | 66(83.54%) |  |
| Pneumonia, n(%) |  |  |  |
| Yes | 65(41.94%) | 25(31.65%) | 0.13 |
| No | 90(58.06%) | 54(68.35%) |  |
| Severe pneumonia, n(%) |  |  |  |
| Yes | 26(16.77%) | 14(17.72%) | 0.86 |
| No | 129(83.23%) | 65(82.28%) |  |
| Cough, n(%) |  |  |  |
| Yes | 139(89.68%) | 71(89.87%) | 0.96 |
| No | 16(10.32%) | 8(10.13%) |  |
| Expectoration, n(%) |  |  |  |
| Yes | 118(76.13%) | 59(74.68%) | 0.81 |
| No | 37(23.87%) | 20(25.32%) |  |
| Chest tightness, n(%) |  |  |  |
| Yes | 64(41.29%) | 37(46.84%) | 0.42 |
| No | 91(58.71%) | 42(53.16%) |  |
| Wheeze, n(%) |  |  |  |
| Yes | 80(51.61%) | 46(58.23%) | 0.34 |
| No | 75(48.39%) | 33(41.77%) |  |
| Fever, n(%) |  |  |  |
| Yes | 95(61.29%) | 48(60.76%) | 0.94 |
| No | 60(38.71%) | 31(39.24%) |  |
| Dyspnea, n(%) |  |  |  |
| Yes | 6(3.87%) | 2(2.53%) | 0.59 |
| No | 149(96.13%) | 77(97.47%) |  |
| Heart failure, n(%) |  |  |  |
| Yes | 3(1.94%) | 1(1.27%) | 1.00 |
| No | 152(98.06%) | 78(98.73%) |  |
| Respiratory failure, n(%) |  |  |  |
| Yes | 62(40.00%) | 28(35.44%) | 0.50 |
| No | 93(60.00%) | 51(64.56%) |  |
| **Comorbidities** |  |  |  |
| History of [valve surgery](https://cncontext.com/english-chinese/translation/valvular+surgery), n(%) |  |  |  |
| Yes | 2(1.29%) | 4(5.06%) | 0.08 |
| No | 153(98.71%) | 75(94.94%) |  |
| Atrial fibrillation, n(%) |  |  |  |
| Yes | 5(3.23%) | 2(2.53%) | 0.77 |
| No | 150(96.77%) | 77(97.47%) |  |
| Coronary Heart Disease, n(%) |  |  |  |
| Yes | 8(5.16%) | 5(6.33%) | 0.71 |
| No | 147(94.84%) | 74(93.67%) |  |
| Cerebral Infarction, n(%) |  |  |  |
| Yes | 16(10.32%) | 7(8.86%) | 0.72 |
| No | 139(89.68%) | 72(91.14%) |  |
| VTE, n(%) |  |  |  |
| Yes | 4(2.58%) | 0(0.00%) | 0.15 |
| No | 151(97.42%) | 79(100.00%) |  |
| Pulmonary embolism, n(%) |  |  |  |
| Yes | 1(0.65%) | 0(0.00%) | 0.47 |
| No | 154(99.35%) | 79(100.00%) |  |
| Hypertension, n(%) |  |  |  |
| Yes | 63(40.65%) | 26(32.91%) | 0.25 |
| No | 92(59.35%) | 53(67.09%) |  |
| Diabetes, n(%) |  |  |  |
| Yes | 33(21.29%) | 16(20.25%) | 0.85 |
| No | 122(78.71%) | 63(79.75%) |  |
| Cardiovascular diseases, n(%) |  |  |  |
| Yes | 69(44.52%) | 29(36.71%) | 0.25 |
| No | 86(55.48%) | 50(63.29%) |  |
| Chronic Respiratory Disease, n(%) |  |  |  |
| Yes | 151(97.42%) | 77(97.47%) | 0.98 |
| No | 4(2.58%) | 2(2.53%) |  |
| Chronic kidney disease, n(%) |  |  |  |
| Yes | 23(14.84%) | 9(11.39%) | 0.47 |
| No | 132(85.16%) | 70(88.61%) |  |
| Digestive system diseases, n(%) |  |  |  |
| Yes | 9(5.81%) | 5(6.33%) | 0.87 |
| No | 146(94.19%) | 74(93.67%) |  |
| Neurological diseases, n(%) |  |  |  |
| Yes | 7(4.52%) | 2(2.53%) | 0.46 |
| No | 148(95.48%) | 77(97.47%) |  |
| Hematological diseases, n(%) |  |  |  |
| Yes | 5(3.23%) | 2(2.53%) | 0.77 |
| No | 150(96.77%) | 77(97.47%) |  |
| Autoimmune diseases, n(%) |  |  |  |
| Yes | 27(17.42%) | 13(16.46%) | 0.85 |
| No | 128(82.58%) | 66(83.54%) |  |
| Types of underlying diseases, mean (sd) | 2.45±1.22 | 2.29±1.26 | 0.35 |
| **Laboratory tests following influenza A and prior to IPA diagnosis** | | |  |
| D-Dimer, mean (sd) | 2.07±3.85 | 2.01±3.4 | 0.91 |
| INR, mean (sd) | 1.08±0.16 | 1.41±3.08 | 0.20 |
| APTT, mean (sd) | 28.69±3.71 | 27.47±4.74 | 0.07 |
| FIB, mean (sd) | 4.42±1.6 | 4.63±2.1 | 0.36 |
| BPC, mean (sd) | 202.2±100.64 | 206.25±96.57 | 0.72 |
| WBC, mean (sd) | 8.05±4.52 | 8.24±3.51 | 0.73 |
| NEU, mean (sd) | 6.33±4.34 | 6.32±3.62 | 0.98 |
| MONO, mean (sd) | 0.61±0.87 | 0.52±0.26 | 0.41 |
| EOS, mean (sd) | 0.33±1.41 | 0.13±0.31 | 0.21 |
| Hb, mean (sd) | 118.16±19.1 | 119.14±22.31 | 0.73 |
| LYM, mean (sd) | 1.14±0.65 | 1.2±0.73 | 0.46 |
| CRP, mean (sd) | 58.85±59.56 | 55.95±50.9 | 0.73 |
| [BLD](http://wap.medix.cn/Module/Examination/ReferenceAndSignificance/Text.aspx?code=02010000012), n(%) |  |  |  |
| Yes | 53(34.19%) | 28(35.44%) | 0.85 |
| No | 102(65.81%) | 51(64.56%) |  |
| FOB, n(%) |  |  |  |
| Yes | 47(30.32%) | 21(26.58%) | 0.55 |
| No | 108(69.68%) | 58(73.42%) |  |
| CD4＜200, n(%) |  |  |  |
| Yes | 29(18.71%) | 11(13.92%) | 0.36 |
| No | 126(81.29%) | 68(86.08%) |  |
| CD4/CD8 ratio＜1, n(%) |  |  |  |
| Yes | 36(23.23%) | 19(24.05%) | 0.89 |
| No | 119(76.77%) | 69(75.95%) |  |
| IgE＞375, n(%) |  |  |  |
| Yes | 8(5.16%) | 4(5.06%) | 0.97 |
| No | 147(94.84%) | 75(94.04%) |  |
| PCT, mean (sd) | 0.62±1.56 | 1.34±5.36 | 0.12 |
| IL-6, mean (sd) | 77.23±185.29 | 76.92±154.37 | 0.99 |
| LDH, mean (sd) | 261.5±101.9 | 270.24±130.68 | 0.57 |
| ALT, mean (sd) | 28.77±45.07 | 33.01±31.02 | 0.44 |
| AST, mean (sd) | 32.42±30.83 | 36.47±42.37 | 0.38 |
| STB, mean (sd) | 10.93±5.75 | 11.83±5.48 | 0.27 |
| Alb, mean (sd) | 34.2±5.02 | 34.66±4.11 | 0.46 |
| Cr, mean (sd) | 73.62±52.59 | 73.48±25.48 | 0.99 |
| Serum and/or BAL GM antigen >1, n(%) |  |  |  |
| Yes | 25(16.13%) | 17(21.52%) | 0.31 |
| No | 130(83.87%) | 62(78.48%) |  |
| (1→3)-β-D-glucan 0–60 pg/mL, n(%) |  |  |  |
| Yes | 127(81.94%) | 69(87.34%) | 0.29 |
| No | 28(18.06%) | 10(12.66%) |  |
| (1→3)-β-D-glucan 60–100 pg/mL, n(%) |  |  |  |
| Yes | 9(5.81%) | 3(3.80%) | 0.73 |
| No | 146(94.19%) | 76(96.20%) |  |
| (1→3)-β-D-glucan >100 pg/mL, n(%) |  |  |  |
| Yes | 19(12.26%) | 7(8.86%) | 0.43 |
| No | 136(87.74%) | 72(91.14%) |  |
| Aspergillus GM antigen >0.5, n(%) |  |  |  |
| Yes | 7(4.52%) | 2(2.53%) | 0.69 |
| No | 148(95.48%) | 77(97.47%) |  |
| Aspergillus-specific IgG 0–79 AU/mL, n(%) |  |  |  |
| Yes | 140(90.32%) | 75(94.94%) | 0.22 |
| No | 15(9.68%) | 4(5.06%) |  |
| Aspergillus-specific IgG 80–119 AU/mL, n(%) |  |  |  |
| Yes | 3(1.94%) | 3(3.80%) | 0.68 |
| No | 152(98.06%) | 76(96.20%) |  |
| Aspergillus-specific IgG >119 AU/mL, n(%) |  |  |  |
| Yes | 12(7.74%) | 1(1.27%) | 0.08 |
| No | 143(92.26%) | 78(98.73%) |  |
| **The use of wide-spectrum antibiotic before IPA diagnose** | |  |  |
| Course of treatment, n(%) |  |  |  |
| <7 days | 21(13.55%) | 14(17.72%) | 0.40 |
| 7-14 days | 59(38.06%) | 29(36.71%) | 0.84 |
| >14 days | 67(43.23%) | 33(41.77%) | 0.83 |
| Cephalosporins, n(%) |  |  |  |
| Yes | 56(36.13%) | 28(35.44%) | 0.92 |
| No | 99(63.87%) | 51(64.56%) |  |
| Fluoroquinolones, n(%) |  |  |  |
| Yes | 91(58.71%) | 38(48.10%) | 0.12 |
| No | 64(41.29%) | 41(51.90%) |  |
| Enzyme inhibitor, n(%) |  |  |  |
| Yes | 89(57.42%) | 43(54.43%) | 0.66 |
| No | 66(42.58%) | 36(45.57%) |  |
| Carbapenem, n(%) |  |  |  |
| Yes | 29(18.71%) | 13(16.46%) | 0.67 |
| No | 126(81.29%) | 66(83.54%) |  |
| Vancomycin, Teicoplanin, Linezolid, Daptomycin, n(%) |  |  |  |
| Yes | 23(14.84%) | 7(8.86%) | 0.20 |
| No | 132(85.16%) | 72(91.14%) |  |
| Special-use antibiotics, n(%) |  |  |  |
| Yes | 14(9.03%) | 5(6.33%) | 0.47 |
| No | 141(90.07%) | 74(93.67%) |  |
| **Glucocorticoids use within three months** | |  |  |
| Cumulative Dose, mean (sd) | 287.11±487.67 | 230.23±354.69 | 0.91 |
| Course of treatment, n(%) |  |  |  |
| ≤7 days | 44(28.39%) | 29(36.71%) | 0.19 |
| 8-14 days | 21(13.55%) | 12(15.19%) | 0.73 |
| 14-28 days | 17(10.97%) | 5(6.33%) | 0.25 |
| >28 days | 14(9.03%) | 7(8.86%) | 0.97 |
| **Other medication before IPA diagnosis** | |  |  |
| The use of Bronchodilator for inhalation (within a month), n(%) |  |  |  |
| Yes | 97(62.58%) | 43(54.43%) | 0.23 |
| No | 58(37.42%) | 36(45.57%) |  |
| The use of ICS (within a month), n(%) |  |  |  |
| Yes | 93(60.00%) | 42(53.16%) | 0.32 |
| No | 62(40.00%) | 37(46.84%) |  |
| Immunosuppressive drugs, n(%) |  |  |  |
| Yes | 20(12.90%) | 10(12.66%) | 0.96 |
| No | 135(87.10%) | 69(87.34%) |  |
| Chemotherapy, n(%) |  |  |  |
| Yes | 23(14.84%) | 15(18.99%) | 0.42 |
| No | 132(85.16%) | 64(81.01%) |  |
| **Imaging examination** |  |  |  |
| Cavity, n(%) |  |  |  |
| Yes | 3(1.94%) | 4(5.06%) | 0.36 |
| No | 152(98.06%) | 75(94.94%) |  |
| Tree-in-bud pattern, n(%) |  |  |  |
| Yes | 34(21.94%) | 18(22.78%) | 0.88 |
| No | 121(78.06%) | 61(77.22%) |  |
| GGO, n(%) |  |  |  |
| Yes | 47(30.32%) | 22(27.85%) | 0.70 |
| No | 108(69.68%) | 57(72.15%) |  |
| Reticular shallow, n(%) |  |  |  |
| Yes | 44(28.39%) | 25(31.65%) | 0.61 |
| No | 111(71.61%) | 54(68.35%) |  |
| Consolidation, n(%) |  |  |  |
| Yes | 4(2.58%) | 2(2.53%) | 1.00 |
| No | 151(97.42%) | 77(97.47%) |  |
| Pleural effusion, n(%) |  |  |  |
| Yes | 58(37.42%) | 23(29.11%) | 0.21 |
| No | 97(62.58%) | 56(70.89%) |  |
| Mediastinal lymphadenectasis, n(%) |  |  |  |
| Yes | 61(39.35%) | 40(50.63%) | 0.10 |
| No | 94(60.65%) | 39(49.37%) |  |
| **Special medical procedures in ICU** |  |  |  |
| ICU admission, n(%) |  |  |  |
| Yes | 36(23.23%) | 16(20.25%) | 0.61 |
| No | 119(76.77%) | 63(79.75%) |  |
| Mechanical ventilation, n(%) |  |  |  |
| Yes | 26(16.77%) | 12(15.19%) | 0.76 |
| No | 129(83.23%) | 67(84.81%) |  |
| Central venous catheter, n(%) |  |  |  |
| Yes | 11(7.10%) | 4(5.06%) | 0.55 |
| No | 144(92.90%) | 75(94.94%) |  |

**Supplementary Table 3. Baseline characteristics of patients in the Training set.**

| Patient characteristics | Con set (n=117) | IPA set (n=38) | P-value |
| --- | --- | --- | --- |
| **Demographics** |  |  |  |
| Age, mean (sd) | 66.68±13.25 | 68.37±8.94 | 0.463 |
| BMI, mean (sd) | 23.12±3.96 | 22.47±4 | 0.056 |
| Gender |  |  |  |
| Male, n (%) | 62(52.99%) | 14(36.84%) | 0.273 |
| Female, n(%) | 55(47.01%) | 24(63.16%) |  |
| **Lifestyle factors, pulmonary comorbidities and respiratory symptoms** | | | |
| Smoking, n(%) |  |  |  |
| Yes | 30(25.64%) | 16(42.11%) | 0.054 |
| No | 87(74.36%) | 22(57.89%) |  |
| Drinking, n (%) |  |  |  |
| Yes | 15(12.82%) | 8(21.05%) | 0.215 |
| No | 102(87.18%) | 30(78.95%) |  |
| Lung cancer, n(%) |  |  |  |
| Yes | 28(23.93%) | 3(7.89%) | 0.032 |
| No | 89(76.07%) | 35(92.11%) |  |
| Interstitial lung disease, n(%) |  |  |  |
| Yes | 29(24.79%) | 18(47.37%) | 0.009 |
| No | 88(75.21%) | 20(52.63%) |  |
| Pulmonary emphysema and bullae lung, n(%) |  |  |  |
| Yes | 23(19.66%) | 11(28.95%) | 0.229 |
| No | 94(80.34%) | 27(71.05%) |  |
| Bronchiectasis, n(%) |  |  |  |
| Yes | 19(16.24%) | 2(5.26%) | 0.086 |
| No | 98(83.76%) | 36(94.74%) |  |
| AECOPD, n(%) |  |  |  |
| Yes | 28(23.93%) | 7(18.42%) | 0.480 |
| No | 89(76.07%) | 31(81.58%) |  |
| Pulmonary hypertension, n(%) |  |  |  |
| Yes | 10(8.55%) | 6(15.79%) | 0.333 |
| No | 107(91.45%) | 32(84.21%) |  |
| Pleural effusion, n(%) |  |  |  |
| Yes | 20(17.09%) | 8(21.05%) | 0.582 |
| No | 97(82.91%) | 30(78.95%) |  |
| pneumonia, n(%) |  |  |  |
| Yes | 50(46.73%) | 15(39.47%) | 0.440 |
| No | 67(53.27%) | 23(60.53%) |  |
| Severe pneumonia, n(%) |  |  |  |
| Yes | 16(13.68%) | 10(26.32%) | 0.070 |
| No | 101(86.32%) | 28(73.68%) |  |
| Cough, n(%) |  |  |  |
| Yes | 102(87.18%) | 37(97.37%) | 0.137 |
| No | 15(12.82%) | 1(2.63%) |  |
| Expectoration, n(%) |  |  |  |
| Yes | 83(70.94%) | 35(92.11%) | 0.008 |
| No | 34(29.06%) | 3(7.89%) |  |
| Chest tightness, n(%) |  |  |  |
| Yes | 43(36.75%) | 21(55.26%) | 0.044 |
| No | 74(63.25%) | 17(44.74%) |  |
| Wheeze, n(%) |  |  |  |
| Yes | 51(43.59%) | 29(76.32%) | <0.001 |
| No | 66(56.41%) | 9(23.68%) |  |
| Fever, n(%) |  |  |  |
| Yes | 75(64.10%) | 20(52.63%) | 0.207 |
| No | 42(35.90%) | 18(47.37%) |  |
| Dyspnea, n(%) |  |  |  |
| Yes | 4(3.42%) | 2(5.26%) | 0.978 |
| No | 113(96.58%) | 36(94.74%) |  |
| Heart failure, n(%) |  |  |  |
| Yes | 3(2.56%) | 0(0.00%) | 0.750 |
| No | 114(97.44%) | 38(100.00%) |  |
| Respiratory failure, n(%) |  |  |  |
| Yes | 45(38.46%) | 17(44.74%) | 0.493 |
| No | 72(61.54%) | 21(55.26%) |  |
| **Comorbidities** |  |  |  |
| History of [valve surgery](https://cncontext.com/english-chinese/translation/valvular+surgery), n(%) |  |  |  |
| Yes | 1(0.85%) | 1(2.63%) | 0.987 |
| No | 116(99.15%) | 37(97.37%) |  |
| Atrial fibrillation, n(%) |  |  |  |
| Yes | 4(3.42%) | 1(2.63%) | 1.000 |
| No | 113(96.58%) | 37(97.37%) |  |
| Coronary Heart Disease, n(%) |  |  |  |
| Yes | 6(5.13%) | 2(5.26%) | 1.000 |
| No | 111(94.87%) | 36(94.74%) |  |
| Cerebral Infarction, n(%) |  |  |  |
| Yes | 12(10.26%) | 4(10.53%) | 1.000 |
| No | 105(89.74%) | 34(89.47%) |  |
| VTE, n(%) |  |  |  |
| Yes | 2(1.71%) | 2(5.26%) | 0.541 |
| No | 115(98.29%) | 36(94.74%) |  |
| Pulmonary embolism, n(%) |  |  |  |
| Yes | 1(0.85%) | 0(0.00%) | 1.000 |
| No | 116(99.15%) | 38(100.00%) |  |
| Hypertension, n(%) |  |  |  |
| Yes | 50(46.73%) | 13(34.21%) | 0.181 |
| No | 67(53.27%) | 25(65.79%) |  |
| Diabetes, n(%) |  |  |  |
| Yes | 21(17.95%) | 12(31.58%) | 0.075 |
| No | 96(82.05%) | 26(68.42%) |  |
| Cardiovascular diseases, n(%) |  |  |  |
| Yes | 50(46.73%) | 19(50.00%) | 0.729 |
| No | 67(53.27%) | 19(50.00%) |  |
| Chronic Respiratory Disease, n(%) |  |  |  |
| Yes | 115(98.29%) | 36(94.74%) | 0.541 |
| No | 2(1.71%) | 2(5.26%) |  |
| Chronic kidney disease, n(%) |  |  |  |
| Yes | 15(12.82%) | 8(21.05%) | 0.215 |
| No | 102(87.18%) | 30(78.95%) |  |
| Digestive system diseases, n(%) |  |  |  |
| Yes | 4(3.42%) | 5(13.16%) | 0.067 |
| No | 113(96.58%) | 33(86.84%) |  |
| Neurological diseases, n(%) |  |  |  |
| Yes | 7(5.98%) | 1(2.63%) | 0.697 |
| No | 110(94.02%) | 37(97.37%) |  |
| Hematological diseases, n(%) |  |  |  |
| Yes | 3(2.56%) | 2(5.26%) | 0.772 |
| No | 114(97.44%) | 36(94.74%) |  |
| Autoimmune diseases, n(%) |  |  |  |
| Yes | 14(11.97%) | 13(34.21%) | 0.002 |
| No | 103(88.03%) | 25(65.79%) |  |
| Types of underlying diseases, mean (sd) | 2.32±1.16 | 2.84±1.35 | 0.040 |
| **Laboratory tests following influenza A and prior to IPA diagnosis** | | |  |
| D-Dimer, mean (sd) | 1.66±3.12 | 3.32±5.36 | 0.020 |
| INR, mean (sd) | 1.09±0.15 | 1.08±0.19 | 0.830 |
| APTT, mean (sd) | 28.8±3.72 | 28.36±3.7 | 0.530 |
| FIB, mean (sd) | 4.26±1.47 | 4.9±1.89 | 0.055 |
| BPC, mean (sd) | 200.08±105.21 | 208.68±86.16 | 0.610 |
| WBC, mean (sd) | 7.64±4.31 | 9.3±4.97 | 0.025 |
| NEU, mean (sd) | 5.81±3.98 | 7.94±5.02 | 0.008 |
| MONO, mean (sd) | 0.59±0.75 | 0.66±1.18 | 0.670 |
| EOS, mean (sd) | 0.27±1.09 | 0.54±2.12 | 0.033 |
| Hb, mean (sd) | 119.82±17.71 | 113.05±22.35 | 0.057 |
| LYM, mean (sd) | 1.24±0.69 | 0.83±0.4 | <0.001 |
| CRP, mean (sd) | 53.64±59.85 | 74.6±56.57 | 0.002 |
| [BLD](http://wap.medix.cn/Module/Examination/ReferenceAndSignificance/Text.aspx?code=02010000012), n(%) |  |  |  |
| Yes | 42(35.90%) | 11(28.95%) | 0.433 |
| No | 75(64.10%) | 27(71.05%) |  |
| FOB, n(%) |  |  |  |
| Yes | 39(33.33%) | 8(21.05%) | 0.152 |
| No | 78(66.67%) | 30(78.95%) |  |
| CD4＜200, n(%) |  |  |  |
| Yes | 15(12.82%) | 14(36.84%) | 0.001 |
| No | 102(87.18%) | 24(63.16%) |  |
| CD4/CD8 ratio＜1, n(%) |  |  |  |
| Yes | 28(23.93%) | 15(39.47%) | 0.063 |
| No | 89(76.07%) | 23(60.53%) |  |
| IgE＞375, n(%) |  |  |  |
| Yes | 5(4.27%) | 3(7.89%) | 0.649 |
| No | 112(95.73%) | 35(92.11%) |  |
| PCT, mean (sd) | 0.52±1.34 | 0.92±2.09 | 0.180 |
| IL-6, mean (sd) | 60.92±98.82 | 126.56±328.06 | 0.058 |
| LDH, mean (sd) | 252.4±93.33 | 289.5±121.85 | 0.051 |
| ALT, mean (sd) | 29.09±48.01 | 27.78±34.93 | 0.890 |
| AST, mean (sd) | 33.3±33.44 | 29.74±21.19 | 0.570 |
| STB, mean (sd) | 10.73±4.82 | 11.51±7.97 | 0.490 |
| Alb, mean (sd) | 34.84±5.01 | 32.26±4.59 | 0.006 |
| Cr, mean (sd) | 68.71±26.48 | 88.5±94.33 | 0.039 |
| Serum and/or BAL GM antigen >1, n(%) |  |  |  |
| Yes | 3(2.56%) | 22(57.89%) | <0.001 |
| No | 114(97.44%) | 16(42.11%) |  |
| (1→3)-β-D-glucan 60–100 pg/mL, n(%) |  |  |  |
| Yes | 19(16.24%) | 9(23.68%) | 0.300 |
| No | 98(83.76%) | 29(76.32%) |  |
| (1→3)-β-D-glucan >100 pg/mL, n(%) |  |  |  |
| Yes | 115(98.29%) | 33(86.84%) | 0.012 |
| No | 2(1.71%) | 5(13.16%) |  |
| Aspergillus GM antigen >0.5, n(%) |  |  |  |
| Yes | 106(90.60%) | 34(89.47%) | 1.000 |
| No | 11(9.40%) | 4(10.53%) |  |
| Aspergillus-specific IgG 80–119 AU/mL, n(%) |  |  |  |
| Yes | 9(7.69%) | 3(7.89%) | 1.000 |
| No | 108(92.31%) | 35(92.11%) |  |
| **The use of wide-spectrum antibiotic before IPA diagnose** | |  |  |
| Course of treatment, n(%) |  |  |  |
| <7 days | 18(15.38%) | 3(7.89%) | 0.241 |
| 7-14 days | 50(42.74%) | 9(23.68%) | 0.036 |
| >14 days | 42(35.90%) | 25(65.79%) | 0.001 |
| Cephalosporins, n(%) |  |  |  |
| Yes | 41(35.04%) | 15(39.47%) | 0.621 |
| No | 76(64.96%) | 23(60.53%) |  |
| Fluoroquinolones, n(%) |  |  |  |
| Yes | 65(55.56%) | 26(68.42%) | 0.162 |
| No | 52(44.44%) | 12(31.58%) |  |
| Enzyme inhibitor, n(%) |  |  |  |
| Yes | 65(55.56%) | 24(63.16%) | 0.410 |
| No | 52(44.44%) | 14(36.84%) |  |
| Carbapenem, n(%) |  |  |  |
| Yes | 18(15.38%) | 11(28.95%) | 0.063 |
| No | 99(84.62%) | 27(71.05%) |  |
| Vancomycin, Teicoplanin, Linezolid, Daptomycin, n(%) |  |  |  |
| Yes | 15(12.82%) | 8(21.05%) | 0.215 |
| No | 102(87.18%) | 30(78.95%) |  |
| Special-use antibiotics, n(%) |  |  |  |
| Yes | 9(7.69%) | 5(13.16%) | 0.487 |
| No | 108(92.31%) | 33(86.84%) |  |
| **Glucocorticoids use within three months** | |  |  |
| Cumulative Dose, mean (sd) | 197.12±358.96 | 564.21±693.86 | <0.001 |
| Course of treatment, n(%) |  |  |  |
| ≤7 days | 35(29.91%) | 9(23.68%) | 0.459 |
| 8–28 days | 20(17.09%) | 18(47.37%) | <0.001 |
| >28 days | 9(7.69%) | 5(13.16%) | 0.487 |
| **Other medication before IPA diagnosis** | |  |  |
| The use of Bronchodilator for inhalation within a month, n(%) |  |  |  |
| Yes | 72(61.54%) | 25(65.79%) | 0.638 |
| No | 45(38.46%) | 13(34.21%) |  |
| The use of ICS within a month, n(%) |  |  |  |
| Yes | 66(56.41%) | 27(71.05%) | 0.109 |
| No | 51(43.59%) | 11(28.95%) |  |
| Immunosuppressive drugs, n(%) |  |  |  |
| Yes | 12(10.26%) | 8(21.05%) | 0.148 |
| No | 105(89.74%) | 30(78.95%) |  |
| Chemotherapy, n(%) |  |  |  |
| Yes | 21(17.95%) | 2(5.26%) | 0.056 |
| No | 96(82.05%) | 36(94.74%) |  |
| **Imaging examination** |  |  |  |
| Cavity, n(%) |  |  |  |
| Yes | 2(1.71%) | 1(2.63%) | 1.000 |
| No | 115(98.29%) | 37(97.37%) |  |
| Tree-in-bud pattern, n(%) |  |  |  |
| Yes | 27(23.08%) | 7(18.42%) | 0.547 |
| No | 90(76.92%) | 31(81.58%) |  |
| GGO, n(%) |  |  |  |
| Yes | 37(31.62%) | 10(26.32%) | 0.536 |
| No | 80(68.38%) | 28(73.68%) |  |
| Reticular shallow, n(%) |  |  |  |
| Yes | 29(24.79%) | 15(39.47%) | 0.081 |
| No | 88(75.21%) | 23(60.53%) |  |
| Consolidation, n(%) |  |  |  |
| Yes | 3(2.56%) | 1(2.63%) | 1.000 |
| No | 114(97.44%) | 37(97.37%) |  |
| Pleural effusion, n(%) |  |  |  |
| Yes | 43(36.75%) | 15(39.47%) | 0.763 |
| No | 74(63.25%) | 23(60.53%) |  |
| Mediastinal lymphadenectasis, n(%) |  |  |  |
| Yes | 44(37.61%) | 17(44.74%) | 0.434 |
| No | 73(62.39%) | 21(55.26%) |  |
| **Special medical procedures in ICU** |  |  |  |
| ICU admission, n(%) |  |  |  |
| Yes | 24(20.51%) | 12(31.58%) | 0.160 |
| No | 93(79.49%) | 26(68.42%) |  |
| Mechanical ventilation, n(%) |  |  |  |
| Yes | 16(13.68%) | 10(26.32%) | 0.070 |
| No | 101(86.32%) | 28(73.68%) |  |
| Central venous catheter, n(%) |  |  |  |
| Yes | 7(5.98%) | 4(10.53%) | 0.559 |
| No | 110(94.02%) | 34(89.47%) |  |

**Supplementary Table 4. Multivariate logistic regression analysis of risk factors for secondary IPA in hospitalized patients with influenza A**

| Variables | β | SE | Wald χ² | *p* value | OR | 95% C.I. for OR | | |
| --- | --- | --- | --- | --- | --- | --- | --- | --- |
|  |  |  |  |  |  | Lower | | Upper |
| Lung cancer | -0.515 | 1.088 | 0.224 | 0.636 | 0.597 | 0.071 | 5.037 | |
| Bronchiectasis | -1.141 | 1.069 | 1.140 | 0.286 | 0.319 | 0.039 | 2.595 | |
| Diabetes | 0.506 | 0.688 | 0.540 | 0.462 | 1.658 | 0.430 | 6.389 | |
| Autoimmune diseases | 1.783 | 0.771 | 5.346 | 0.021 | 5.946 | 1.312 | 26.947 | |
| Smoking | 2.376 | 0.761 | 9.740 | 0.002 | 10.758 | 2.420 | 47.826 | |
| D-Dimer | 0.029 | 0.067 | 0.184 | 0.668 | 1.029 | 0.903 | 1.172 | |
| Fibrinogen | 0.524 | 0.236 | 4.922 | 0.027 | 1.688 | 1.063 | 2.681 | |
| EOS | 0.320 | 0.246 | 1.692 | 0.193 | 1.378 | 0.850 | 2.232 | |
| Hb | -0.041 | 0.019 | 4.822 | 0.028 | 0.960 | 0.925 | 0.996 | |
| LYM | -1.634 | 0.754 | 4.694 | 0.030 | 0.195 | 0.045 | 0.856 | |
| CRP | -0.006 | 0.007 | 0.887 | 0.346 | 0.994 | 0.980 | 1.007 | |
| CD4/CD8 ratio＜1 | 0.003 | 0.690 | 0.000 | 0.997 | 1.003 | 0.259 | 3.880 | |
| PCT | -0.058 | 0.216 | 0.072 | 0.789 | 0.944 | 0.619 | 1.440 | |
| IL-6 | 0.000 | 0.002 | 0.025 | 0.874 | 1.000 | 0.996 | 1.004 | |
| Alb | -0.040 | 0.071 | 0.314 | 0.575 | 0.961 | 0.836 | 1.105 | |
| Course of wide-spectrum antibacterial treatment >14 days | 0.625 | 0.688 | 0.826 | 0.363 | 1.869 | 0.485 | 7.198 | |
| Fluoroquinolones | 1.252 | 0.648 | 3.739 | 0.053 | 3.497 | 0.983 | 12.443 | |
| Carbapenem | -0.659 | 0.785 | 0.705 | 0.401 | 0.517 | 0.111 | 2.408 | |
| Cumulative GC Dose | 0.002 | 0.001 | 5.613 | 0.018 | 1.002 | 1.000 | 1.003 | |
| GC treatment Course of 8–28 days | 1.627 | 0.632 | 6.635 | 0.010 | 5.090 | 1.476 | 17.556 | |
| The use of ICS (within a month) | 0.818 | 0.630 | 1.686 | 0.194 | 2.265 | 0.659 | 7.782 | |
| Chemotherapy | -2.202 | 1.531 | 2.070 | 0.150 | 0.111 | 0.006 | 2.221 | |
| Mechanical ventilation | -0.859 | 0.782 | 1.205 | 0.272 | 0.424 | 0.091 | 1.963 | |

**Abbreviations table of the main text**

| AI | Artificial intelligence |
| --- | --- |
| AUC | Area under the receiver operating characteristic curve |
| BMI | Body mass index |
| EMR | Electronic Medical Record |
| EORTC | 2020 European Organization for Research on Treatment of Cancer |
| G test | (1→3)-β-D-glucan test |
| GC | Glucocorticoid |
| GM test | Galactomannan test |
| GSCV | Grid Search Cross-Validation |
| Hb | Hemoglobin |
| IAPA | Influenza-associated invasive pulmonary aspergillosis |
| ICU | Intensive Care Uite |
| IL-6 | Interleukin-6 |
| IPA | Invasive pulmonary aspergillosis |
| LightGBM | Light Gradient Boosting Machine |
| LR | Logistic Regression |
| LYM | Lymphocyte count |
| MSG | Mycology Study Group |
| NPV | Negative prediction value |
| OR | Odds ratios |
| PCT | Procalcitonin |
| PPV | Positive prediction value |
| RF | Random Forest |
| ROC | Receiver operating characteristic |
| SD | Standard deviation |
| SE | Standard error |
| SHAP | SHapley Additive exPlanations |
| SVM | Support Vector Machine |
| Wald χ² | Wald chi-square test statistic |
| XGBoost | Extreme Gradient Boosting |
| β | β Coefficient |

**Supplementary table of abbreviations**

| AECOPD | Acute exacerbations of chronic obstructive pulmonary disease |
| --- | --- |
| Alb | Albumin |
| ALT | Alanine Aminotransferase |
| APTT | Activated partial thromboplastin time |
| AST | Aspartate Aminotransferase |
| [BLD](http://wap.medix.cn/Module/Examination/ReferenceAndSignificance/Text.aspx?code=02010000012) | Urine Occult Blood |
| BPC | Blood Platelet Count |
| Cr | Creatinine |
| CRP | C-reactive Protein |
| EOS | Eosinophil Count |
| FIB | Fibrinogen |
| FOB | Feces Occult Blood |
| GC | Glucocorticoids |
| GGO | Ground-glass opacity |
| Hb | Hemoglobin |
| ICS | Inhaled Corticosteroids |
| IL-6 | Interleukin-6 |
| INR | International Normalized Ratio |
| LDH | Lactate Dehydrogenase |
| LY% | Lymphocyte Percentage |
| LYM | Lymphocyte Count |
| MONO | Monocyte Count |
| NEU | Neutrophil Count |
| NEUT% | Neutrophil Percentage |
| PCT | Procalcitonin |
| STB | Serum Total Bilirubin |
| VTE | Venous thromboembolism |
| WBC | White Blood Cell Count |
